# Clinical Characteristics, Imaging Findings and Outcomes in Neonatal Septic Arthritis: A Tertiary NICU Experience

**DOI:** 10.3390/children13040495

**Published:** 2026-04-01

**Authors:** Hatice Turgut, Ramazan Ozdemir

**Affiliations:** Department of Pediatrics, Division of Neonatology, Faculty of Medicine, Inonu University, Malatya 44280, Türkiye

**Keywords:** newborn, magnetic resonance imaging, osteomyelitis, septic arthritis, *Staphylococcus aureus*

## Abstract

**Highlights:**

**What are the main findings?**

- Neonatal septic arthritis frequently presents with subtle clinical features and is associated with high rates of concomitant osteomyelitis (5/12) and early orthopedic sequelae (6/12).
- Ultrasonography failed to detect joint pathology in 67% of cases, whereas MRI consistently revealed inflammatory changes consistent with septic arthritis.

**What are the implications of the main findings?**

- Normal ultrasonographic findings should not exclude the diagnosis when clinical suspicion persists in neonates.
- Early recognition and timely management are critical to reducing the risk of osteomyelitis and long-term sequelae.

**Abstract:**

Background: Neonatal septic arthritis is a rare but potentially devastating infection that can present with subtle clinical signs and rapidly progress to joint destruction, osteomyelitis, and permanent sequelae. We aimed to describe the clinical and microbiological characteristics, imaging findings, management, and follow-up outcomes of neonatal septic arthritis patients and to report ultrasonography (US) and magnetic resonance imaging (MRI) findings within the same cohort. Methods: This retrospective observational study was conducted in a tertiary neonatal intensive care unit and included neonates who were diagnosed with septic arthritis between January 2016 and December 2025. Demographic, clinical, laboratory, microbiological, imaging, treatment, and outcome data were systematically obtained from medical records. The diagnosis was based on compatible clinical findings supported by laboratory and/or microbiological evidence, with imaging used to support diagnosis and guide management. Results: Twelve neonates were included. The median gestational age was 36.5 weeks (26–40), and the median birth weight was 2435 g. The median symptom onset was 22 days of life. The hip (n = 4) and knee (n = 4) were most commonly affected. Pseudoparalysis and swelling were the most frequent findings. Synovial cultures were positive in 10/12 (*Staphylococcus aureus*, n = 7; methicillin-resistant *Staphylococcus aureus* (MRSA), n = 3), and blood cultures were positive in 6/12. US detected joint effusion in 33% of the patients, whereas MRI revealed inflammatory changes in all the patients. Concomitant osteomyelitis occurred in 5/12 patients. Orthopedic sequelae developed in 6/12; no mortality was observed. Conclusions: Neonatal septic arthritis is associated with a substantial risk of osteomyelitis and early sequelae. Delayed recognition may worsen outcomes. Normal ultrasonographic findings should not exclude the diagnosis when clinical suspicion persists, and MRI may provide complementary information for timely management.

## 1. Introduction

Septic arthritis is a rare condition in neonates; however, delays in diagnosis and treatment can lead to substantial morbidity and mortality. In neonates, bacterial microorganisms can readily gain access to the joint space because of immune system immaturity, the lax structure of the joint capsule, and the presence of vascular connections between the metaphysis and epiphysis. Consequently, neonatal septic arthritis may rapidly progress to joint destruction, osteomyelitis and permanent sequelae [1,2,3].

Although the true incidence of neonatal septic arthritis is unclear, various reports have estimated that it ranges from 0.12 to 3 cases per 1000 live births [4,5]. The hip and knee are the joints most frequently involved, and establishing an early diagnosis is often challenging. Diagnosis is often delayed because clinical manifestations are nonspecific and may be obscured by concomitant sepsis. In addition, joint examination in neonates is technically challenging, physical signs can be subtle, and early radiographic findings may not clearly indicate infection. Therefore, a high index of clinical suspicion is essential for timely recognition and management [2,3,6].

Ultrasonography (US) is frequently used as a first-line imaging modality because it is noninvasive and can be performed at the bedside. However, its diagnostic yield may be limited in the early stages of infection or in patients with concomitant osteomyelitis. Magnetic resonance imaging (MRI) provides superior visualization of intra-articular and periarticular structures and may facilitate early detection of bone involvement, although its use may be limited by availability, cost, and the need for sedation [7].

Although numerous studies on septic arthritis in childhood are available in the literature, evidence specific to the neonatal period remains limited, and a substantial proportion of publications consist of single case reports or small case series. Existing studies show marked heterogeneity with respect to culture positivity rates, pathogen distribution, duration of antibiotic therapy, need for surgical intervention, and clinical outcomes. Nevertheless, data on early sequelae rates in neonatal septic arthritis patients and the clinical implications of imaging findings remain scarce.

In this context, the present study aimed to characterize the clinical presentation, microbiological profile, imaging findings, management approaches, and follow-up outcomes of neonates with septic arthritis treated in a tertiary neonatal intensive care unit. By examining ultrasonography and magnetic resonance imaging findings within the same cohort, we sought to offer clinically relevant insights into the use of imaging in neonatal septic arthritis patients.

## 2. Materials and Methods

### 2.1. Study Design and Setting

This retrospective single-center case series was conducted in a tertiary neonatal intensive care unit and included neonates diagnosed and managed for septic arthritis between January 2016 and December 2025. The relevant demographic, clinical, laboratory, imaging, and treatment data were retrieved from medical records.

### 2.2. Patient Population

Neonates who were diagnosed with and managed for septic arthritis in the neonatal intensive care unit during the study period were eligible for inclusion. Cases were identified through a review of medical records. Patients whose essential clinical data were incomplete were excluded from the analysis.

The diagnosis of septic arthritis was established on the basis of compatible clinical findings supported by laboratory and/or microbiological evidence. Imaging modalities, including ultrasonography (US) and magnetic resonance imaging (MRI), were used to support clinical assessment and to evaluate the extent of joint and bone involvement. Imaging findings were not considered determinative on their own but contributed to the overall clinical decision-making process. The final assessment was made on the basis of the patients’ clinical course and a comprehensive evaluation of all available findings.

### 2.3. Data Collection and Variables

Patient medical records were reviewed retrospectively. The following data were extracted: demographic characteristics (gestational age, birth weight, sex, and mode of delivery); clinical variables (time to diagnosis, presence of fever and accompanying clinical and physical examination findings, and the affected joint); laboratory parameters (white blood cell count, neutrophil and lymphocyte counts, hemoglobin, and platelet values; infection markers [CRP and IL-6]; blood culture and synovial fluid culture results); and treatment-related variables (type and duration of antibiotic therapy and the need for surgical intervention). In addition, potential risk factors, including neonatal intensive care unit (NICU) hospitalization, prematurity, perinatal asphyxia, sepsis, anemia, and a history of central venous catheterization, were recorded. Clinical outcomes, including morbidity and mortality, were also documented.

### 2.4. Treatment Protocol

Empirical antibiotic therapy was initiated according to the standard protocol for our unit (a third-generation cephalosporin plus an aminoglycoside or an aminoglycoside plus vancomycin) and subsequently adjusted on the basis of culture results and clinical response.

Patients with significant joint effusion, inadequate clinical response to medical therapy, or imaging findings suggestive of osteomyelitis were evaluated for joint aspiration and/or surgical drainage.

The duration of antibiotic therapy was determined according to clinical evolution and the presence of osteomyelitis, which generally ranged between 4 and 6 weeks.

### 2.5. Sample Size

As this study was conducted as a descriptive case series, no formal sample size calculation was performed. All consecutive eligible cases identified during the study period were included in the analysis.

### 2.6. Statistical Analysis

Statistical analyses were performed using SPSS version 26.0 (IBM Corp., Armonk, NY, USA). Continuous variables are expressed as the mean ± standard deviation or median (minimum–maximum), as appropriate. Categorical variables are presented as frequencies and percentages. Given the descriptive nature and limited sample size of the study, the analyses were restricted to descriptive statistics.

### 2.7. Ethics Approval and Consent to Participate

This study was conducted in accordance with the ethical principles of the Declaration of Helsinki (1975, revised in 2013). Ethical approval was obtained from the Institutional Scientific Research and Publication Ethics Committee (Approval No. 2025/9128; Date: 13 January 2026).

Given the retrospective and noninterventional design of the study, the requirement for informed consent was waived by the Ethics Committee. All patient data were anonymized before analysis, and confidentiality was strictly maintained throughout the research process.

## 3. Results

A total of 12 neonates diagnosed with septic arthritis were included in the study (Table 1). The median gestational age was 36.5 weeks (range, 26–40), and the median birth weight was 2435 g (range, 520–3800). The median age at diagnosis was 22 days (range, 3–60 days), and the median interval between diagnosis and surgical intervention was 11 days (range, 4–17 days). The sex distribution included five males and seven females. Eight infants were delivered by cesarean section, and four were delivered vaginally. The most commonly affected joints were the hip (n = 4) and knee (n = 4), followed by the shoulder (n = 2) and elbow (n = 2).

Among the clinical findings, the most common presentations were pseudoparalysis (n = 10) and swelling (n = 10). Fever was detected in only one patient. Pain and increased local warmth were present in four patients. Hemoglobin levels ranged from 7.4 to 13.3 g/dL, platelet counts ranged from 110,000 to 907,000/mm^3^, and white blood cell counts ranged from 3700 to 7500/mm^3^. Neutrophil predominance was observed in all the infants. CRP levels were elevated in all patients (2.9–19.7 mg/L).

IL-6 levels were measured in six patients and were elevated in all patients. As summarized in Table 2, the reported risk factors included NICU hospitalization before the onset of osteoarticular infection, perinatal asphyxia, anemia, sepsis, prematurity, and a history of central venous catheterization. In this cohort, 9 of the 12 patients (75.0%) were admitted to the NICU before symptom onset, whereas 3 patients presented from the outpatient setting with pseudoparalysis and/or localized swelling.

When the microbiological findings were evaluated, *Staphylococcus aureus* was isolated from the synovial fluid cultures of seven patients, and methicillin-resistant *Staphylococcus aureus* (MRSA) was detected in three patients, whereas no growth was detected in two patients. In concurrently obtained blood cultures, MRSA was identified in two patients, and *S. aureus* was identified in four patients.

At presentation, plain radiographs were obtained for all patients to exclude fractures. During follow-up, all the infants underwent joint US and contrast-enhanced MRI. On US, the findings were reported as normal in eight patients, whereas joint effusion was detected in four patients. In contrast, contrast enhancement was observed on MRI in all patients. Concomitant osteomyelitis was identified in five patients (41.7%). All patients underwent surgical management with joint aspiration and/or drainage. Empirical antibiotic therapy was subsequently revised according to the culture and antimicrobial susceptibility test results. The duration of antibiotic therapy ranged from 28 to 42 days, and treatment was extended to 42 days in eight patients.

During follow-up, six infants developed clinically significant orthopedic sequelae, including limitation of motion at the hip or shoulder, joint dysplasia or deformity, subluxation, and gait disturbance. Because the study spanned a 10-year period, the total follow-up duration varied across patients, and follow-up is still ongoing for some infants diagnosed in the later years of the study. Therefore, orthopedic outcomes were assessed on the basis of a standardized minimum of 1 year of postinfection follow-up.

No mortality was observed in this case series.

## 4. Discussion

Although neonatal septic arthritis is rare, it is a severe infection characterized by rapid joint destruction and the potential to cause permanent injury to the epiphysis and growth plate. Several factors, including prematurity, low birth weight, prolonged NICU hospitalization, invasive catheterization, anemia, mechanical ventilation, perinatal asphyxia, and concomitant neonatal sepsis, have been identified in the literature as key determinants of the development of neonatal septic arthritis [1,8]. In preterm infants, insufficient immunological maturation increases susceptibility to infection, whereas prolonged hospitalization and invasive procedures increase the risk of colonization with nosocomial pathogens and subsequent bacteremia. Berberian and colleagues reported that 69% of their patients had at least one infection-related risk factor, most of which were associated with nosocomial infections [3]. Sreenivas and colleagues reported that the main risk factors for developing neonatal septic arthritis were prematurity (69%), sepsis (55%), asphyxia (51%), and anemia (89.7%) [6]. Rubin et al. further suggested that invasive practices, including parenteral nutrition, surgical procedures, and mechanical ventilatory support, may also constitute important risk factors [8]. In our cohort, the presence of at least two of the risk factors previously described in the literature supports the multifactorial etiology of neonatal septic arthritis. It is plausible that the coexistence of multiple risk factors in the same patient increases the infectious burden, facilitates hematogenous seeding of the joint during bacteremia, and contributes to a more severe clinical course.

In our case series, the most frequently observed clinical findings were pseudoparalysis and local swelling. Fever was present in only one patient. In contrast, Sreenivas et al. reported a more pronounced clinical presentation, with fever observed in all cases (100%) and pseudoparalysis (69.0%), swelling (79.3%), and increased local warmth (75.9%) among the most common findings [6]. Conversely, in a multicenter study including 45 cases, swelling was reported in 64%, erythema in 56%, and pseudoparalysis in 22% of patients [9]. These discrepancies suggest that clinical manifestations may vary substantially depending on patient characteristics and factors such as the extent of infection and the timing of diagnosis.

In a study in which preterm infants constituted the majority (69%), the mean age at symptom onset for septic arthritis was reported to be 23.8 ± 4.3 days [6]. In another study, which included an even greater percentage of preterm infants (87%), the time to symptom onset was 30 days (IQR 15–48) [9]. In a cohort with a preterm birth rate of 13%, symptoms were reported to begin at 17.5 ± 7.6 days [10]. In our series, 41.7% of the infants were preterm, and the median age at symptom onset was 22 days (range, 3–60 days). Taken together, these data suggest that symptoms of septic arthritis tend to emerge later in preterm infants. In our cohort, the lower rate of prematurity compared with that in prior studies may have contributed to earlier recognition of clinical signs and, consequently, a relatively earlier reported age at symptom onset. In preterm infants, factors such as delayed immune responses, increased risk of colonization with nosocomial pathogens, and prolonged NICU hospitalization may influence the timing of infection and a predisposition to later symptom development. Conversely, in term infants, more rapid evolution of clinical manifestations may facilitate earlier detection of infection.

In the case series reported by Lee et al., the mean interval between presentation and surgical intervention was 15.2 days (range, 4–25 days), and 83% of the patients (n = 5) experienced a complicated recovery. The authors emphasized that one of the main reasons for this delay was false-negative culture results. They also reported that an undetectable collection in aspirated joint or periarticular fluid or the absence of bacterial growth on culture does not exclude the diagnosis of septic arthritis [11].

Similarly, delayed intervention has been identified as a potentially modifiable factor influencing clinical outcomes. Delays exceeding seven days have been reported to be associated with poor prognosis, underscoring the importance of early recognition and timely surgical management [12]. These observations support the notion that, particularly in the neonatal period—when clinical and laboratory findings may be subtle—management decisions should rely on sustained clinical suspicion rather than awaiting definitive culture confirmation.

In our study, the interval between presentation and surgical intervention was 10.6 ± 4.3 days (median 11; range 4–17 days), and 50% of the patients developed sequelae. The interval to surgical intervention likely reflects a combination of diagnostic, clinical, and logistical factors, including the limited diagnostic yield of initial ultrasonography in some cases, the need for MRI in selected patients, the stabilization of clinically fragile neonates, and perioperative planning. Although the time to surgery was shorter than that reported in the literature, the persistently high rate of sequelae underscores the critical importance of early diagnosis and timely surgical intervention in neonatal septic arthritis. Early surgical drainage is essential for reducing intra-articular pressure, lowering the risk of epiphyseal ischemia, and decreasing the likelihood of permanent joint damage [9]. In addition, diagnostic challenges, such as a low initial level of clinical suspicion, nonspecific or equivocal early imaging findings, and culture negativity, may further increase the risk of sequelae.

Although microbiological confirmation is key in the diagnosis of septic arthritis, culture positivity is often limited in neonates because of physiological and clinical factors unique to this age group. In the literature, positivity rates for blood cultures and other bacteriological tests range from 52% to 82% [1]. Sreenivas and colleagues also highlighted this variability, reporting positivity rates of 17% for blood cultures and 62% for synovial fluid cultures [6]. This wide range is thought to be associated with several clinical and technical factors, including the frequent initiation of antibiotic therapy before cultures are obtained, a low bacterial burden, and infection presenting at an early or localized stage.

A similar pattern was observed in our study; 50% of blood cultures and 83% of synovial fluid cultures were positive. These findings indicate that culture negativity does not exclude the diagnosis of neonatal septic arthritis; rather, a diagnosis should be made through an integrated assessment of clinical findings, laboratory parameters, and imaging modalities. Moreover, the isolation of the same pathogen from synovial fluid in four of the five patients with positive blood cultures supports the concept that neonatal septic arthritis is most commonly a hematogenously disseminated infection.

When the distribution of causative pathogens was examined, *Staphylococcus aureus* was reported as the most frequently isolated organism in neonatal septic arthritis in the majority of the literature, which is consistent with our findings. In a multicenter study by Rubin et al., methicillin-susceptible *S. aureus* (MSSA) and methicillin-resistant *S. aureus* (MRSA) were identified in 71% and 18% of cases, respectively, clearly demonstrating the predominant role of *S. aureus* in neonatal infections [9]. Similarly, Li et al. reported an *S. aureus* isolation rate of 64.7% [10]. In contrast, Uthraraj et al. reported MSSA in 18% and gram-negative pathogens in 82% of cases [5]. Such discrepancies may be explained by variations in each center’s nosocomial flora, antibiotic prescribing practices, clinical characteristics of the patient population and sample size. Overall, these findings indicate that *S. aureus* remains the dominant pathogen in neonatal septic arthritis patients, but intercenter variability should be considered. Therefore, local microbiological resistance patterns and institutional flora should be carefully considered when empirical antibiotic therapy is selected.

The hip joint is particularly vulnerable to infection and prone to rapid destruction and secondary osteomyelitis because of its distinctive vascular architecture during development and relatively limited epiphyseal blood flow. Consequently, hip involvement is frequently highlighted in the literature as the predominant site of infection in neonatal septic arthritis. In a review encompassing 16 studies, hip joint involvement was reported in 42% of cases, whereas knee involvement occurred in 27% [8]. Similarly, in a study evaluating 24 pediatric cases, the hip was affected in 62% of cases, followed by the knee in 24% [13]. In the series reported by Kabak et al., hip and knee involvement were identified in 42% and 36% of cases, respectively, further confirming that the hip was the primary site of involvement [1].

The literature indicates that the hip joint is not only involved more frequently but also has a higher risk of sequelae [12]. In a study by Li et al., the recovery rate without sequelae was reported to be only 33.3% for hip involvement, whereas markedly higher rates were observed for the knee/ankle joints (85.0%) and the shoulder/elbow joints (78.9%). In the same study, hip joint involvement was identified as a significant predictor of an approximately 12-fold increased risk of sequelae [10].

In our study, the equal involvement rates of the hip (33.3%) and knee (33.3%) joints differ somewhat from reports in which hip involvement is typically predominant. Nevertheless, our findings regarding complication development are consistent with the literature. The 50% rates of both sequelae and osteomyelitis in the hip joint support the notion that, owing to its anatomical and vascular characteristics, the hip is more susceptible to infection-related structural damage. More notably, the observation of 100% sequelae among patients with shoulder involvement suggests that, although shoulder involvement is rare, infection at this site may follow a particularly aggressive course.

Although mortality due to neonatal septic arthritis is reported to be low in most studies despite appropriate treatment, it is well recognized that the disease can lead to permanent long-term sequelae. Damage to the growth plate can result in persistent joint disorders and skeletal developmental abnormalities, which can cause orthopedic problems such as limited motion, deformity, subluxation, and gait disturbances. The literature reports sequelae rates ranging from 6% to 50% [3,4,14]. In our case series, 50% of patients developed orthopedic sequelae during follow-up, a rate that is consistent with previously reported outcomes.

US is often the first imaging modality for the diagnosis of septic arthritis because of its low cost and noninvasive nature. Although it is highly sensitive for detecting joint effusion, it provides limited information regarding the underlying cause of the effusion [15]. In the literature, effusion detection rates by ultrasonography have been reported to be 35%, 59.2%, and 75.9% [4,6,9]. Akash Rai et al. emphasized that abnormal findings on initial US may indicate more advanced disease [4]. Conversely, in pediatric patients, US performed within the first 24 h after symptom onset has been associated with a 5% false-negative rate [16]. In a pediatric cohort study, US alone was performed in 67% of the 33 patients diagnosed with septic arthritis of the hip, and treatment was completed without the need for additional imaging [15].

In our series, joint effusion was detected on US at presentation in 33% of the patients. In contrast, the presence of contrast enhancement on MRI in all patients suggests that MRI can detect early inflammatory changes, such as synovitis, effusion, and bone involvement, with increased sensitivity.

In neonates in particular, given the rapid progression of septic arthritis and the risk of permanent sequelae, the cost–benefit balance of early MRI use should be evaluated carefully. In this age group, MRI may offer a clear advantage in preventing diagnostic delay; however, the necessity of its routine use in every case remains debatable in terms of balancing clinical benefit against economic burden. In our case series, MRI was performed in all patients and proved informative for both diagnosis and surgical decision-making. Our findings suggest that MRI may play a valuable complementary role in this patient population.

The primary strength of this study is the detailed characterization of the clinical presentation, microbiological profile, imaging findings, and follow-up outcomes of neonatal septic arthritis, a condition for which published data remain limited. By reporting ultrasonography and magnetic resonance imaging findings within the same cohort, the study offers practical clinical insight into imaging use in this rare population.

Several limitations should be acknowledged. First, the small sample size reflects the rarity of neonatal septic arthritis and limits the generalizability of the findings. Second, the retrospective single-center design may introduce selection bias and carry the risk of incomplete clinical documentation. Third, the absence of standardized long-term functional follow-up restricts conclusions regarding long-term functional outcomes. Finally, given the descriptive nature of the study, no inferential statistical analyses were performed.

Despite these limitations, the findings contribute to clinical awareness of neonatal septic arthritis and highlight the need for larger, prospective, multicenter investigations to better define optimal diagnostic and management strategies.

## 5. Conclusions

Overall, neonatal septic arthritis is a serious infection that may present without prominent early clinical signs but can rapidly progress to joint destruction and permanent sequelae. In our cohort, pseudoparalysis and local swelling were the most common clinical manifestations, and *Staphylococcus aureus* was the predominant pathogen. Magnetic resonance imaging was useful for detecting intra-articular and periarticular involvement, particularly when clinical suspicion persisted despite inconclusive ultrasonographic findings.

The high rates of concomitant osteomyelitis and early sequelae observed in this series highlight the potential consequences of delayed diagnosis and treatment. Early clinical recognition, appropriate antimicrobial therapy, and timely intervention when indicated remain essential for reducing morbidity. Larger prospective, multicenter studies are needed to clarify diagnostic approaches and management strategies for neonatal septic arthritis.

## Figures and Tables

**Table 1:** Case-level characteristics and management of neonatal septic arthritis (n = 12).

Patient	Sex	Delivery	BW (Gram)	GA (Week)	DOL at Onset	Diagnosis–Surgery (Day)	Joint	OM	Blood Cx	Joint Cx	US	MRI	Sequelae	Antibiotic Duration (Day)
1	Male	C/S	2100	32	20	8	Knee	No	MRSA	MRSA	Normal	Positive	No	28
2	Female	C/S	2770	40	24	10	Hip	Yes	No growth	*S. aureus*	Normal	Positive	No	42
3	Male	C/S	3600	37	15	15	Knee	No	No growth	*S. aureus*	Normal	Positive	No	42
4	Female	Vaginal	1900	32	25	17	Hip	Yes	*S. aureus*	*S. aureus*	Normal	Positive	Yes	42
5	Female	C/S	3300	39	15	13	Shoulder	No	No growth	*S. aureus*	Normal	Positive	Yes	30
6	Male	Vaginal	3260	39	16	12	Hip	No	No growth	*S. aureus*	Normal	Positive	Yes	42
7	Female	C/S	1100	28	24	4	Knee	No	*S. aureus*	No growth	Normal	Positive	No	28
8	Male	Vaginal	3800	38	3	15	Elbow	Yes	MRSA	MRSA	Positive	Positive	Yes	42
9	Male	Vaginal	2000	37	16	6	Hip	No	*S. aureus*	*S. aureus*	Positive	Positive	No	30
10	Female	C/S	520	26	60	9	Elbow	No	No growth	MRSA	Positive	Positive	No	42
11	Female	C/S	3500	38	25	5	Shoulder	Yes	No growth	*S. aureus*	Positive	Positive	Yes	42
12	Female	C/S	1030	29	51	14	Knee	Yes	*S. aureus*	No growth	Normal	Positive	Yes	42

Abbreviations: C/S: cesarean section; BW: birth weight; GA: gestational age; DOL: day of life; OM: osteomyelitis; Cx: culture; US: ultrasonography; MRI: magnetic resonance imaging; MRSA: methicillin-resistant *Staphylococcus aureus*; *S. aureus*: *Staphylococcus aureus*. Imaging: ‘Positive’ indicates findings consistent with septic arthritis.

**Table 2:** Distribution of risk factors among patients.

Risk Factors	n (%)
NICU hospitalization	9 (75.0)
Anemia	8 (66.7)
Sepsis	7 (58.3)
Prematurity	5 (41.7)
Central venous catheter	5 (41.7)
Perinatal asphyxia	3 (25.0)

Note: Overlapping risk factors were common in this cohort, with seven patients having two concurrent risk factors and five having three or more concurrent risk factors. NICU: neonatal intensive care.

## Data Availability

The data presented in this study are available on request from the corresponding author. The data are not publicly available for privacy and ethical reasons.

## Abbreviations

The following abbreviations are used in this manuscript:

US	Ultrasonography
NICU	Neonatal intensive care unit
MRSA	Methicillin-resistant *Staphylococcus aureus*
MSSA	Methicillin-susceptible *Staphylococcus aureus*
C/S	Cesarean section
BW	Birth weight
GA	Gestational age
DOL	Day of life
OM	Osteomyelitis
Cx	Culture
MRI	Magnetic resonance imaging
